# Transcriptome analysis of *Crossostephium chinensis* provides insight into the molecular basis of salinity stress responses

**DOI:** 10.1371/journal.pone.0187124

**Published:** 2017-11-13

**Authors:** Haiyan Yang, Ming Sun, Shuangji Lin, Yanhong Guo, Yongjuan Yang, Tengxun Zhang, Jingxing Zhang

**Affiliations:** 1 College of Landscape Architecture, Beijing Forestry University, Beijing, China; 2 Beijing Key Laboratory of Ornamental Plant Germplasm Innovation & Molecular Breeding, Beijing, China; 3 Beijing Laboratory of Urban and Rural Ecological Environment, Beijing, China; 4 National Engineering Research Center for Floriculture, Beijing, China; National Botanical Research Institute CSIR, INDIA

## Abstract

Soil salinization is becoming a limitation to the utilization of ornamental plants worldwide. *Crossostephium chinensis* (Linnaeus) Makino is often cultivated along the southeast coast of China for its desirable ornamental qualities and high salt tolerance. However, little is known about the genomic background of the salt tolerance mechanism in *C*. *chinensis*. In the present study, we used Illumina paired-end sequencing to systematically investigate leaf transcriptomes derived from *C*. *chinensis* seedlings grown under normal conditions and under salt stress. A total of 105,473,004 bp of reads were assembled into 163,046 unigenes, of which 65,839 (40.38% of the total) and 54,342 (33.32% of the total) were aligned in Swiss-Prot and Nr protein, respectively. A total of 11,331 (6.95%) differentially expressed genes (DEGs) were identified among three comparisons, including 2,239 in ‘ST3 vs ST0’, 5,880 in ‘ST9 vs ST3’ and 9,718 in ‘ST9 vs ST0’, and they were generally classified into 26 Gene Ontology terms and 58 Kyoto Encyclopedia of Genes and Genomes (KEGG) pathway terms. Many genes encoding important transcription factors (e.g., *WRKY*, *MYB*, and *AP2/EREBP*) and proteins involved in starch and sucrose metabolism, arginine and proline metabolism, plant hormone signal transduction, amino acid biosynthesis, plant-pathogen interactions and carbohydrate metabolism, among others, were substantially up-regulated under salt stress. These genes represent important candidates for studying the salt-response mechanism and molecular biology of *C*. *chinensis* and its relatives. Our findings provide a genomic sequence resource for functional genetic assignments in *C*. *chinensis*. These transcriptome datasets will help elucidate the molecular mechanisms responsible for salt-stress tolerance in *C*. *chinensis* and facilitate the breeding of new stress-tolerant cultivars for high-saline areas using this valuable genetic resource.

## Introduction

Soil salinization is a major global environmental problem. Approximately one-third of the irrigated land worldwide has been affected by salinized soil, especially in arid and semi-arid regions [1, 2]. In China, *C*. *chinensis* (Linnaeus) Makino is rare and threatened in the wild but is often cultivated on the southeast coast due to its desirable ornamental qualities and high salt tolerance [3, 4]. Additionally, as a relative of *Chrysanthemum*, *C*. *chinensis* is an important germplasm for salt tolerance improvement in *Chrysanthemum* [5]. Therefore, the identification and exploration of the mechanisms underlying salt tolerance in *C*. *chinensis* are required for genotype improvement in *Chrysanthemum*.

Plants have acquired many biochemical and molecular mechanisms to adapt to abiotic stress. Physiological parameters, including proline content [6], superoxide dismutase (SOD) and peroxidase (POD) activity [7], as well as the K^+^ /Na^+^ ratio [8], are important indicators of injury in plants exposed to a saline environment. However, the molecular mechanisms are far more complex. Plant salt-responsive genes can be classified into two groups, those that directly protect plants against environmental stresses and those that regulate the expression of downstream target genes in the stress response [9]. The former group includes various osmoprotectants, late-embryogenesis-abundant (LEA) proteins, aquaporin (APQ) proteins, chaperones, and antioxidant enzymes. The latter group primarily consists of transcription factors, such as *NAC*, *WRKY*, *MYB*, *bZIP*, *ERF*, and *bHLH* transcription factors, which are activated by a series of signal transduction pathways. Additionally, all responsive behavior requires a signaling sensor, signal transduction, transcriptional regulation, gene expression and reactions leading to the production of relevant compounds to reach the final ionic and osmotic balance [10, 11]. Additionally, the abscisic acid (ABA), Ca^2+^-dependent, SOS, and MAPK signaling pathways play important roles in the salt-response process [12–15].

Studies examining salt tolerance and transcriptome-wide surveys in *Chrysanthemum lavandulifolium* and *Chrysanthemum nankingense* have been conducted [16, 17]. In our earlier studies, we found that *Crossostephium chinensis* had stronger resistance to salt stress than did *Chrysanthemum* species. However, no transcriptome information concerning the salt responsiveness of *Crossostephium chinensis* has been reported to date. In this study, the transcriptional sequencing and analysis of *C*. *chinensis* under normal conditions and salt stress were performed using Illumina assembly technology (NEBNext^®^ Ultra^™^ RNA Library Prep Kit, NEB, USA) and RNA-seq quantification analysis. First, we identified differentially expressed genes (DEGs) between the normal sample and the stressed samples. We also identified important responsive pathways and genes involved in salt tolerance in *C*. *chinensis*. These results further contribute to the study of salt-responsive mechanisms and molecular biology in *Chrysanthemum* and its relatives.

## Results

### Physiological parameters

**Fig 1:**
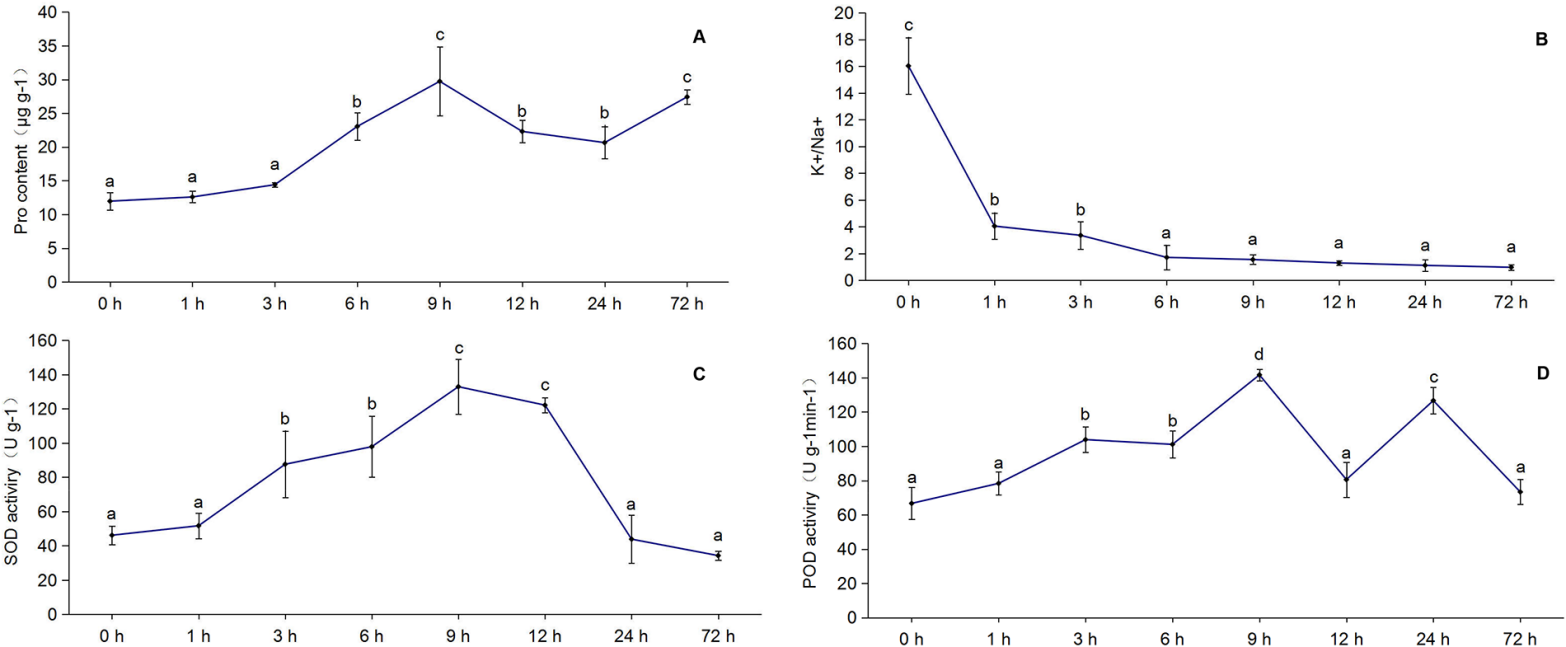
Changes in the physiological parameters of *C*. *chinensis* leaves during NaCl stress. A, B, C, and D show the changes in proline content, the K+/Na+ ratio, SOD activity, and POD activity, respectively, after 0, 1, 3, 6, 9, 12, 24, and 72 hours of 360 mM NaCl stress. Values in each column with the same letter are not significantly different (p = 0.05) as described by Duncan’s test.

The four physiological parameters of *C*. *chinensis* seedlings tended to differ in their variation under 0, 1, 3, 6, 9, 12, 24 and 72 hours of salt stress (Fig 1). There were no significant changes in proline content during the first 3 hours of stress (Fig 1A). A maximum peak occurred at 9 hours, with an increase of 15.31 μg g^-1^ over that at 3 hours. Although it declined at 12 and 24 hours (but was still higher than at time 0), the proline content increased to 15.45 μg g^-1^ more than that of the control at 72 hours. The activity of SOD increased initially and had a peak value of 132.93 U g^-1^ at 9 hours (Fig 1C). After a slight decline at 12 hours, at 24 hours, there was a sharp drop to its lowest activity, which was 2.29 U g^-1^ lower than that in the control. The activity of POD displayed large increases of 37.22 U g^-1^ min^-1^ and 75 U g^-1^ min^-1^ at 3 and 9 hours, respectively, compared with that of the control (Fig 1D). Another peak was observed at 24 hours and was only 15 U g^-1^ min^-1^ less than the value at 9 hours. The activity subsequently declined to the control level of 66.67 U g^-1^ min^-1^. Regarding the K^+^/Na^+^ ratio, the value declined with prolonged stress (Fig 1D). The ratio remained high during the first 3 hours of stress and was greater than 1 at 24 hours, which indicated a greater concentration of K^+^ than of Na^+^.

### Illumina sequencing and assembly

Plant samples grown under the stress of 360 mM NaCl for 0 (ST0), 3 (ST3) and 9 (ST9) hours were selected to study the high-salinity responses of *C*. *chinensis* using the Illumina HiSeq 4000 system platform. Thus, nine cDNA libraries were constructed. The overall sequencing results are shown in Table 1. Stringent quality checks and data cleaning provided 573,602,460 (86.04 G) clean reads. All error rates were less than 0.1%. The general base quality value reached 30%. The GC value ranged from 40% to 50%. These results indicated high-quality sequencing and the feasibility of the subsequent analyses.

**Table 1:** Summary of read statistics from RNA-sequencing of *Crossostephium chinensis*.

Sample	Raw reads	Clean reads	Clean bases	Error (%)	Q20 (%)	Q30 (%)	GC (%)
ST0-1	65,813,022	63,430,918	9.51 G	0.03	94.42	86.74	43.10
ST0-2	69,408,202	66,751,218	10.01 G	0.03	94.70	87.34	43.36
ST0-3	81,457,120	78,293,106	11.74 G	0.03	94.43	86.85	43.26
ST3-1	67,562,116	59,933,628	8.99 G	0.03	95.34	87.57	43.77
ST3-2	66,182,504	63,475,934	9.52 G	0.03	93.53	85.06	43.32
ST3-3	72,085,618	63,570,756	9.54 G	0.03	94.56	86.04	43.73
ST9-1	60,970,456	58,770,710	8.82 G	0.03	94.04	85.91	42.83
ST9-2	65,768,746	63,378,252	9.51 G	0.03	94.27	86.29	43.23
ST9-3	57,970,426	55,997,938	8.4 G	0.03	94.06	85.77	43.89

Low-quality reads were removed using an in-house Perl script. Finally, a total of 163,046 high-quality unigenes with a length of 105,473,004 bp and N50 length of 1,063 bp were retrieved, with an average length of 647 bp, maximum length of 15,593 bp and minimum length of 201 bp. The length distribution of the transcripts and unigenes is shown in S1 Fig.

### Gene annotation and functional classification

To obtain comprehensive gene function information, the unigenes were annotated using seven databases. The concrete annotation results are shown in Table 2. Of the 163,046 unigenes, 65,839 (40.38% of the total) and 54,342 (33.32% of the total) were aligned in Swiss-Prot and Nr protein, respectively, which were the two databases with the most unigene annotations.

**Table 2:** Unigene annotation results from seven databases.

Database	Number of unigenes	Percentage (%)	E-value
Nr	54,342	33.32	1e-5
NT	52,868	32.42	1e-5
KO	37,417	22.94	0.01
Swiss-Prot	65,839	40.38	1e-3
PFAM	52,488	32.19	1e-5
GO	53,368	32.73	1e-10
KOG	34,183	20.96	1e-6
All databases	10,867	6.66	\
At least one database	92,911	56.98	\
Total unigenes	163,046	100	\

The international standardized gene functional classification system Gene Ontology (GO) provides three ontologies—molecular function, cellular component and biological process—that were useful for gene annotation and analysis. Based on the Nr annotation and Swiss-Prot protein databases, 53,368 unigenes were classified into 47 functional GO categories using Blast2GO software [18]. A total of 127,314 unigenes were classified into 23 biochemical process categories, 82,003 unigenes were classified into 19 cellular component categories, and 63,264 unigenes were classified into 14 molecular function categories (S2 Fig). A total of 53.40% and 50.98% of unigenes were classified into ‘cell process’ and ‘metabolic process’, respectively, in ‘biological process’; 30.41% and 30.39% of unigenes were classified into ‘cell’ and ‘cell part’, respectively, in ‘cell component’; and 54.74% and 43.52% of the unigenes were classified into ‘binding’ and ‘catalytic activity’, respectively in ‘molecular function’.

A eukaryote-specific version of the clusters of orthologous groups (COG) tool, euKaryotic Orthologous Groups (KOG), is used to identify orthologous and paralogous proteins, providing a way to identify Joint Genome Institute (JGI)-predicted genes based on the KOG classification or ID. The annotated sequences were further searched to identify genes involved in KOG classifications to evaluate the completeness of our transcriptome library and the effectiveness of our annotation process. Of 54,342 Nr hits, 34,183 sequences were assigned to KOG classifications. Among the 26 KOG categories, the cluster for ‘general function prediction only’ (5,582, 16.33%) represented the largest group, followed by ‘posttranslational modification, protein turnover, chaperones’ (4,290, 12.55%) and ‘signal transduction mechanisms’ (4,100, 11.99%). In contrast, the ‘nuclear structure’ (173, 0.51%), ‘cell motility’ (51, 0.15%) and ‘unnamed protein’ (3, 0.01%) categories represented the smallest groups (S3 Fig).

To further analyze the transcriptome, all unigenes were compared with the Kyoto Encyclopedia of Genes and Genomes (KEGG) database using BLASTx with an E-value threshold of < 1e^-10^ [19]. Of the 163,046 unigenes, 37,417 had significant matches in the database and were assigned to 285 KEGG pathways, which were categorized into four sub-groups as follows: ‘cellular processes’, ‘environmental information processing’, ‘genetic information processing’ and ‘organismal systems’. The most highly represented pathway was ‘signal transduction’ (4,668), followed by ‘translation’ (2,926), ‘folding, sorting and degradation’ (2,545), ‘carbohydrate metabolism’ (2,464), ‘endocrine system’ (2,270), and ‘transport and catabolism’ (2,142) (S4 Fig). These annotated pathways provided us with valuable information for elucidating the response of *C*. *chinensis* to salt stress.

### General expression patterns of DEGs

**Fig 2:**
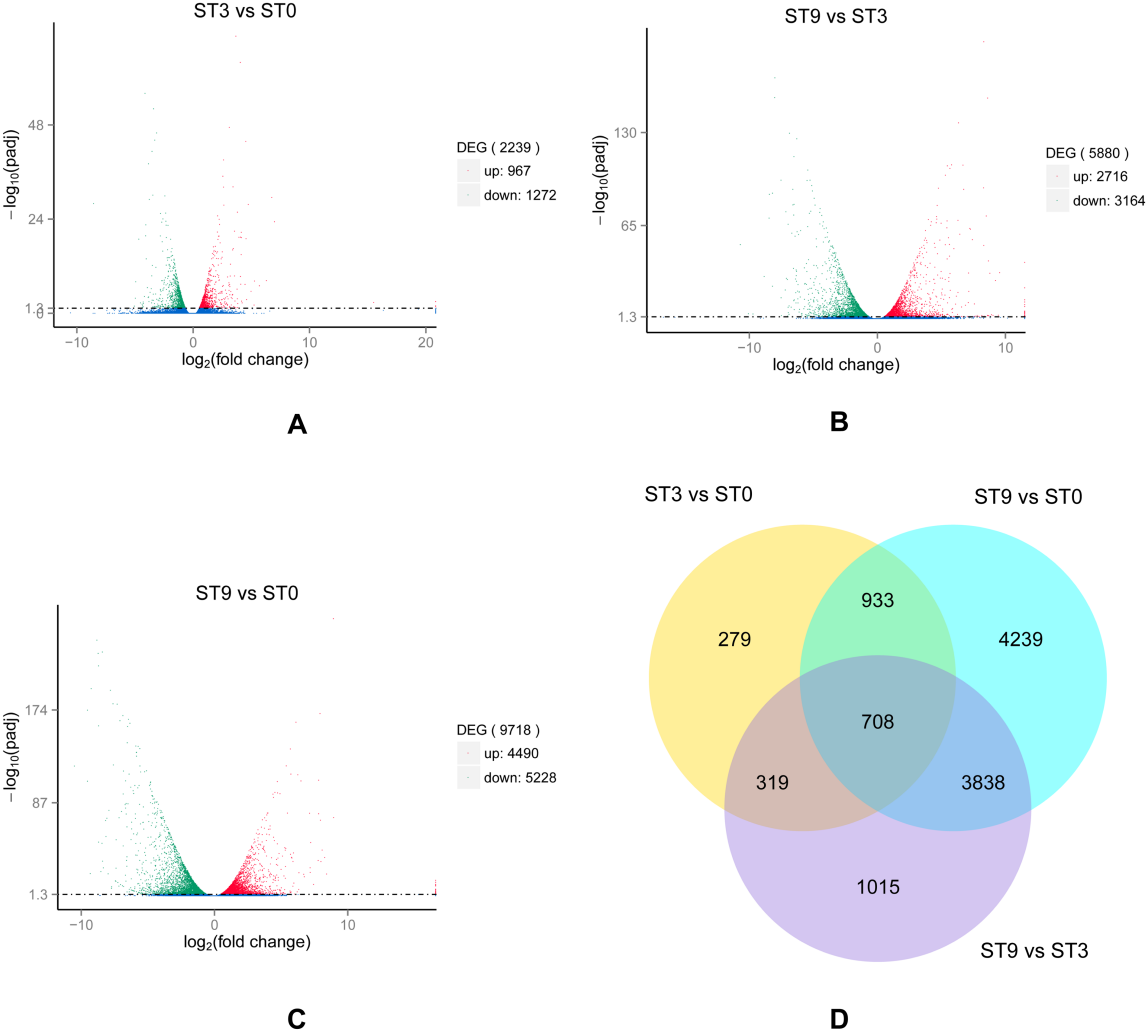
Differential expression patterns of all unigenes among three libraries (ST0, ST3 and ST9). A, B, and C show volcano plot analyses of DEGs in ‘ST3 vs ST0’, ‘ST9 vs ST3’ and ‘ST9 vs ST0’, respectively. The x-axis indicates the expression ratio of the different samples, and the y-axis indicates the significance of the differential gene expression, which is positive in relation to the -log_10_(padj) value and negative in relation to the padj value. Red plots represent up-regulated genes; green plots represent down-regulated genes; and blue plots represent no significant difference. D indicates the number of DEGs shared among different groups.

For transcriptomes with no reference genome, genes were expressed with a value of fragments per kilobase of transcript sequence per million base pairs sequenced (FPKM) > 0.3. For the samples with biological replicates, genes with a padj < 0.05 found by DESeq were considered differentially expressed. A total of 163,046 unigenes in the nine libraries met the criterion of padj < 0.05. We screened 11,331 (6.95%) DEGs, with 2,239 in ‘ST3 vs ST0’ (967 up- and 1,272 down-regulated) (Fig 2A), 5,880 in ‘ST9 vs ST3’ (2,716 up- and 3,164 down-regulated) (Fig 2B), and 9,718 in ‘ST9 vs ST0’ (4,490 up- and 5,228 down-regulated) (Fig 2C). As shown in Fig 2D, some DEGs were present in more than one comparison: seven hundred and eight genes were differentially expressed in all three comparisons.

**Fig 3:**
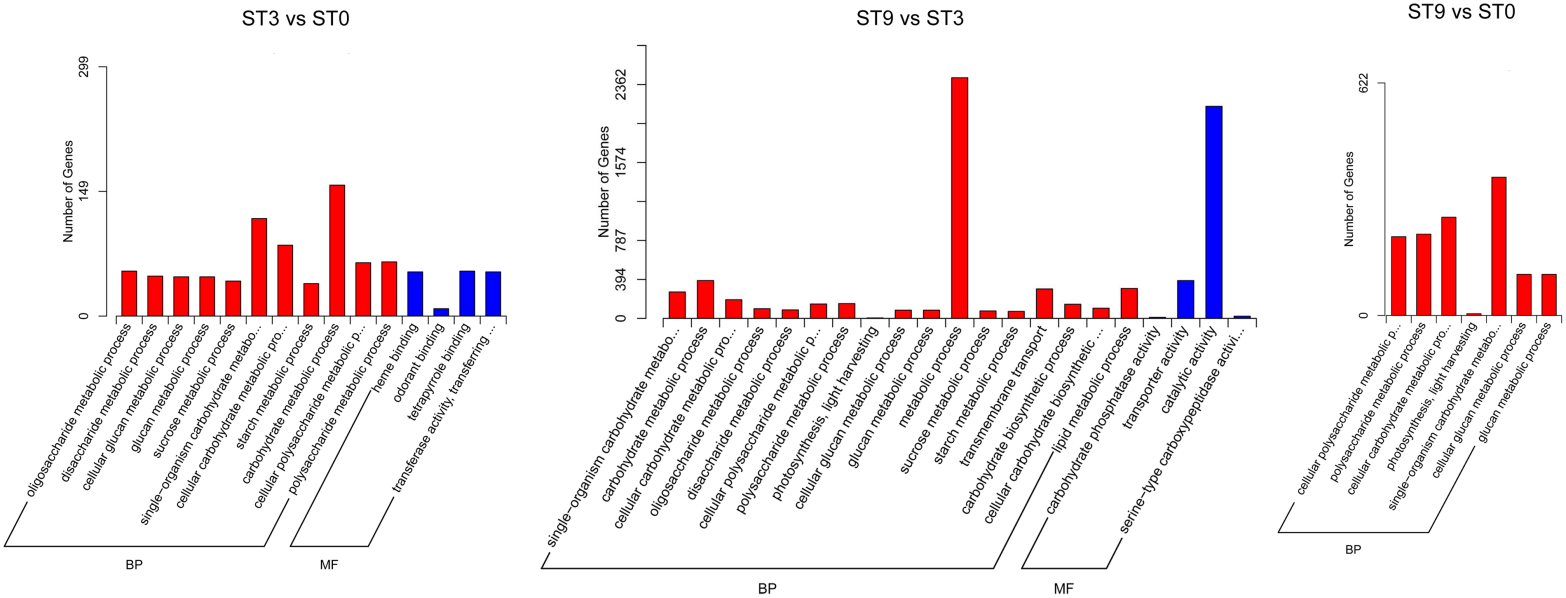
GO enrichment of DEGs in the three compared groups. DEGs in ‘ST3 vs ST0’, ‘ST9 vs ST3’, and ‘ST9 vs ST0’ were enriched for 15, 21, and 7 GO terms, respectively.

**Fig 4:**
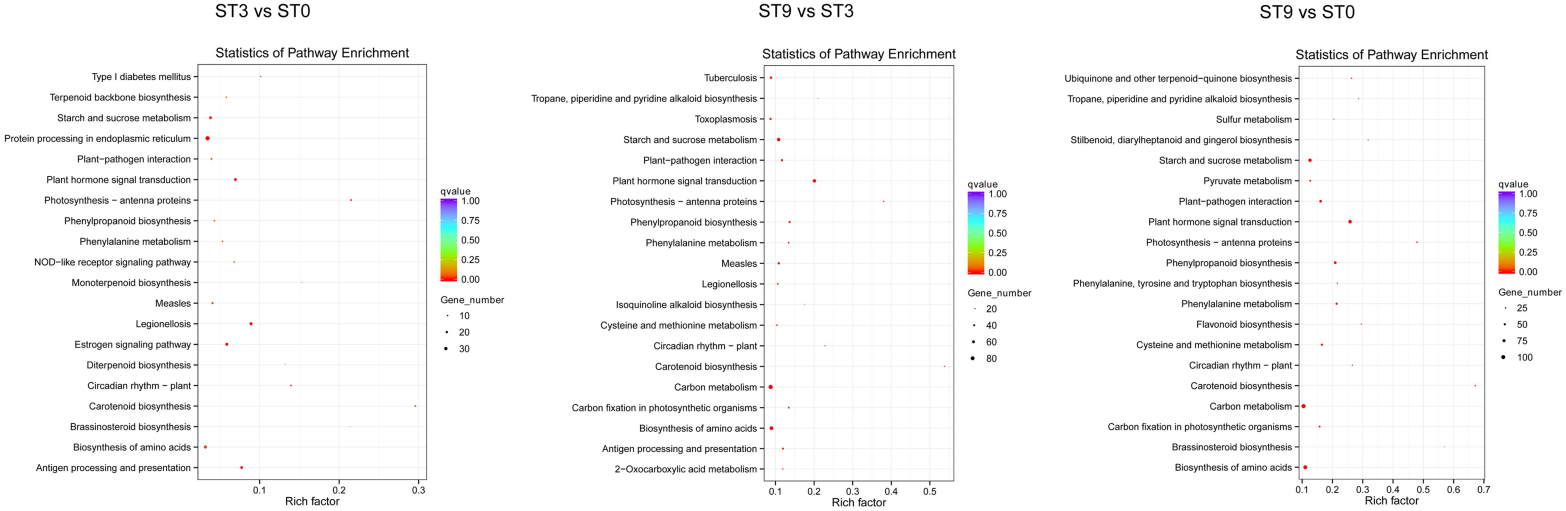
Top 20 enriched KEGG pathways among DEGs from ‘ST3 vs ST0’, ‘ST9 vs ST3’, and ‘ST9 vs ST0’. The number of DEGs in each pathway is positively related to the size of the plots. The padj values shown in red are positive.

**Fig 5:**
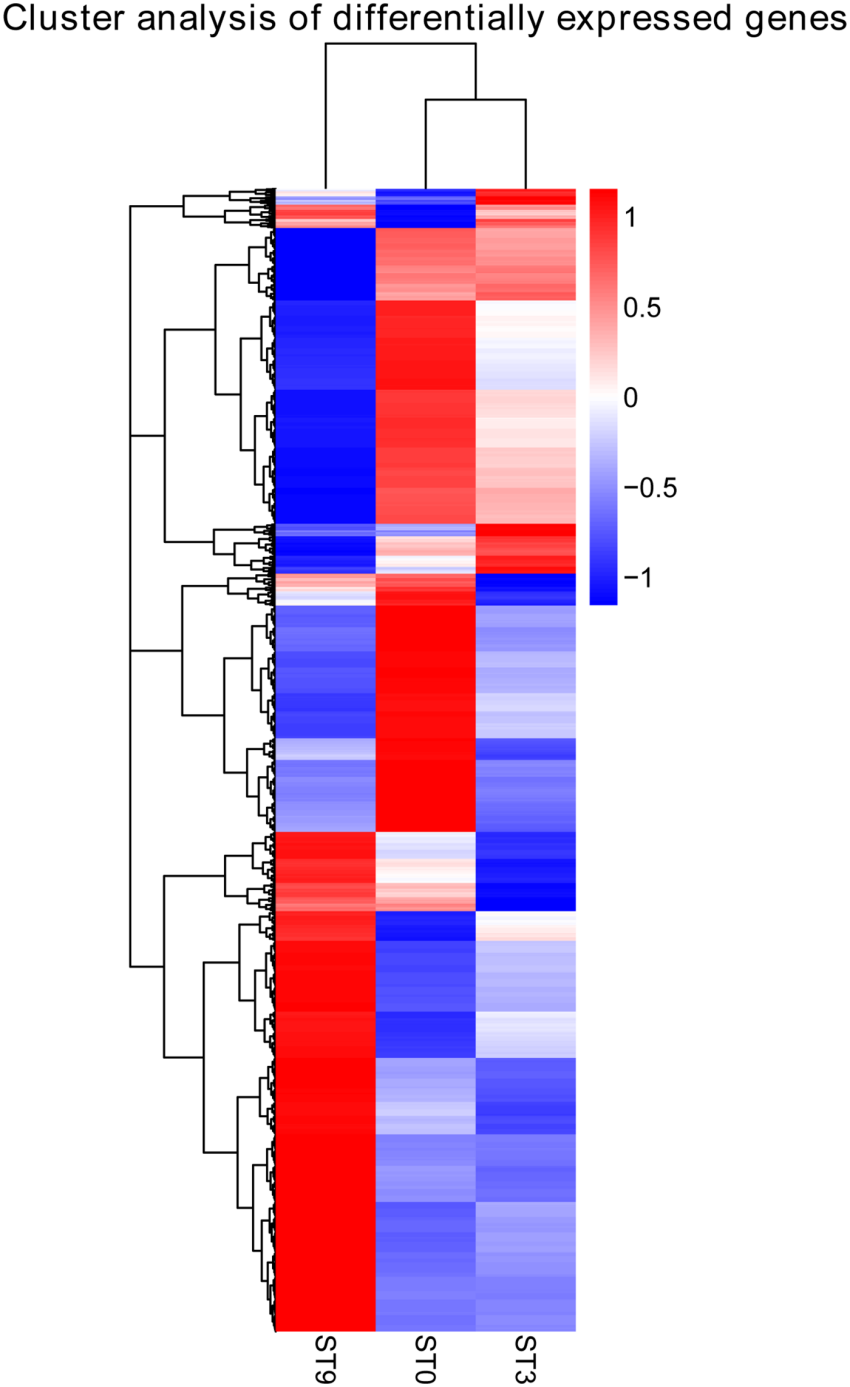
Hierarchical cluster analyses of the 11,331 DEGs in the three samples. Columns and rows in the heat maps represent samples and genes, respectively. Sample names are displayed below the heat maps. The color scale indicates the fold-change in gene expression. Red indicates high expression, and blue indicates low expression.

To further identify differences in biological processes and pathways between the control and salt treatment samples, GO and KEGG pathway enrichment analyses (padj < 0.05) and cluster analysis of DEGs were conducted. Generally, all the GO-annotated DEGs—1,494 (66.73%) in ‘ST3 vs ST0’, 3936 (66.94%) in ‘ST9 vs ST3’, and 6,220 (64.00%) in ‘ST9 vs ST0’—were enriched in 26 terms (S2 Table), among which ‘metabolic process’ and ‘catalytic activity’ were enriched by as many as 2,430 (61.74%) and 2,141 (54.40%), respectively, in ‘ST9 vs ST3’. Additionally, six GO terms related to carbohydrate-metabolism-process-enriched DEGs were found in the three comparisons; these terms included ‘single-organism carbohydrate metabolic process’, ‘cellular polysaccharide metabolic process’, ‘cellular glucan metabolic process’, and ‘glucan metabolic process’, ‘cellular polysaccharide metabolic process’, and ‘polysaccharide metabolic process’, indicating an important role in the *C*. *chinensis* response to salt. Furthermore, 10 GO-term-enriched DEGs were detected in ‘ST9 vs ST3’, while only 5 GO-term-enriched DEGs were found in ‘ST3 vs ST0’, demonstrating that more biological processes were affected after 3 hours of salt stress. Fig 3 shows the GO enrichment results for the three comparisons. All the KEGG Orthology (KO)-annotated DEGs were enriched in 56 KEGG pathway terms (S3 Table). Fig 4 shows the results for the 20 most significantly enriched pathways. For the KEGG pathway analysis, the dominant pathways were as follows: ‘plant hormone signal transduction’, ‘starch and sucrose metabolism’, ‘biosynthesis of amino acids’, ‘phenylpropanoid biosynthesis’, ‘plant-pathogen interaction’, and ‘carbon metabolism’. Cluster analysis was used to determine the expression model of samples from different treatments, as shown in Fig 5.

### Identification of genes that respond to salinity

Salt-responsive processes are involved in a complicated gene regulatory network. Table 3 provides a summary of the changes in some model pathways that play important roles in salt stress in many species. All eight salt-responsive pathways in 31 signal transduction pathways of ‘environmental information processing’ sub-groups included DEGs, among which ‘plant hormone signal transduction’ and ‘PI3K-Akt signaling pathway’ had the most DEGs in the three comparisons (ST3 vs ST0, ST9 vs ST3, and ST9 vs ST0). Additionally, more DEGs were present in the ‘starch and sucrose metabolism’ and ‘arginine and proline metabolism’ sub-groups of ‘metabolism’. The same was observed for DEGs in the ‘plant-pathogen interaction’ group. Pathways that were significantly enriched with DEGs are shown in S3 Table.

**Table 3:** Expression of DEGs in salt-responsive KEGG pathways.

Salt-tolerance-related pathways	ST3 vs ST0		ST9 vs ST3		ST9 vs ST0	
	DOWN	UP	DOWN	UP	DOWN	UP
Arginine and proline metabolism	4	6	17	11	24	13
Glycine, serine and threonine metabolism	3	5	19	11	19	10
Flavonoid biosynthesis	1	1	8	3	18	1
Nitrogen metabolism	0	2	6	2	7	1
Starch and sucrose metabolism	14	11	32	38	36	46
Oxidative phosphorylation	7	3	11	26	26	11
Plant-pathogen interaction	5	10	29	15	37	24
ABC transporters	0	0	5	3	5	4
Plant hormone signal transduction	18	6	38	31	57	32
Calcium signaling pathway	2	3	16	6	23	5
MAPK signaling pathway	2	13	23	3	3	5
FOXO signaling pathway	4	1	8	9	14	10
AMPK signaling pathway	11	3	16	15	28	10
PI3K-Akt signaling pathway	8	9	18	21	39	19
Phosphatidylinositol signaling system	2	4	14	5	17	6

NaCl stress induces DEGs in plants exposed to adverse conditions by inducing the transcriptional expression of the corresponding gene products. These products are generally classified as regulatory proteins and functional proteins. The former participate in signal transduction or gene regulation in response to a stimulus, while the latter function directly in stress-response reactions. Table 4 shows the expression profiles of salt-responsive genes that function in signal transduction, osmotic regulation, ion transduction, functional proteins, and transcription regulation. Genes that participate in the ABA, MAPK, SOS, and Ca^2+^ signal transduction pathways were clearly up- or down-regulated. *PRODH*, *P5CS*, and *OAT* are three key genes in the proline metabolism pathway. *PRODH* and *P5CS* were up- and down-regulated, respectively. In contrast, *OAT* included 2 up-regulated and 1 down-regulated unigenes. Genes related to the ROS response system, *AQPs*, and *LEAs* were more commonly down-regulated, especially *TIP* (2 up- and 5 down-regulated), *PIP* (3 up- and 13 down-regulated) and *AQP* genes. Excluding *BADH* and zinc-fingers, many more genes related to other transcription factors, such as *NAC*, *WRKY*, *MYB*, *bZIP*, *ERF*, and *bHLH*, were differentially expressed.

**Table 4:** Expression of salt-responsive gene families.

Gene name or pathway	Number of genes	Up-regulation	Down-regulation
ABA signaling pathway			
*PP2C*	18	0	12
*PYR/PYL*	13	1	2
*SnRK2*	15	2	3
*ABRE/ABF*	7	2	2
MAPK signaling pathway			
*PPP3C*	21	2	5
*MAPK*	162	7	7
*MAPK1_3*	44	3	1
*HSPA1_8*	96	13	17
Ca^2+^ signaling-related			
*CaM*	35	2	7
*CML*	41	1	11
*CDPK*	52	9	2
SOS signaling pathway			
*HAK*	11	0	1
*HKT*	6	0	2
*AKT*	10	0	3
V-type H^+^-ATPase	133	10	9
Plasma membrane H^+^-ATPase	7	1	1
*NHX*	17	0	3
*CBL (CIPK)*	46	3	23
Proline metabolism			
*PRODH*	9	1	0
*OAT*	2	2	1
*P5CS*	19	0	4
ROS response system			
*SOD*	25	1	1
*GST*	97	5	17
*APX*	11	2	3
*GPX*	23	0	2
*POD*	123	8	12
Functional proteins			
*TIP*	13	2	5
*PIP*	28	3	13
*NIP*	12	0	5
*SIP*	5	0	0
*Other AQPs*	15	0	0
*LEA*	13	2	3
Transcription factors			
*NAC*	103	4	12
*MYB*	125	7	18
*DREB (AP2-EREBP)*	26	5	3
*ERF (AP2-EREBP)*	101	14	16
*WRKY*	89	10	2
*BADH*	6	0	0
*bZIP*	263	20	26
*Zinc-finger*	17	2	2
*bHLH*	106	11	16

The number of genes that were up- and down-regulated compared with that of the control was counted based on the criterion of padj < 0.05.

### Validation of DEGs by qRT-PCR

**Fig 6:**
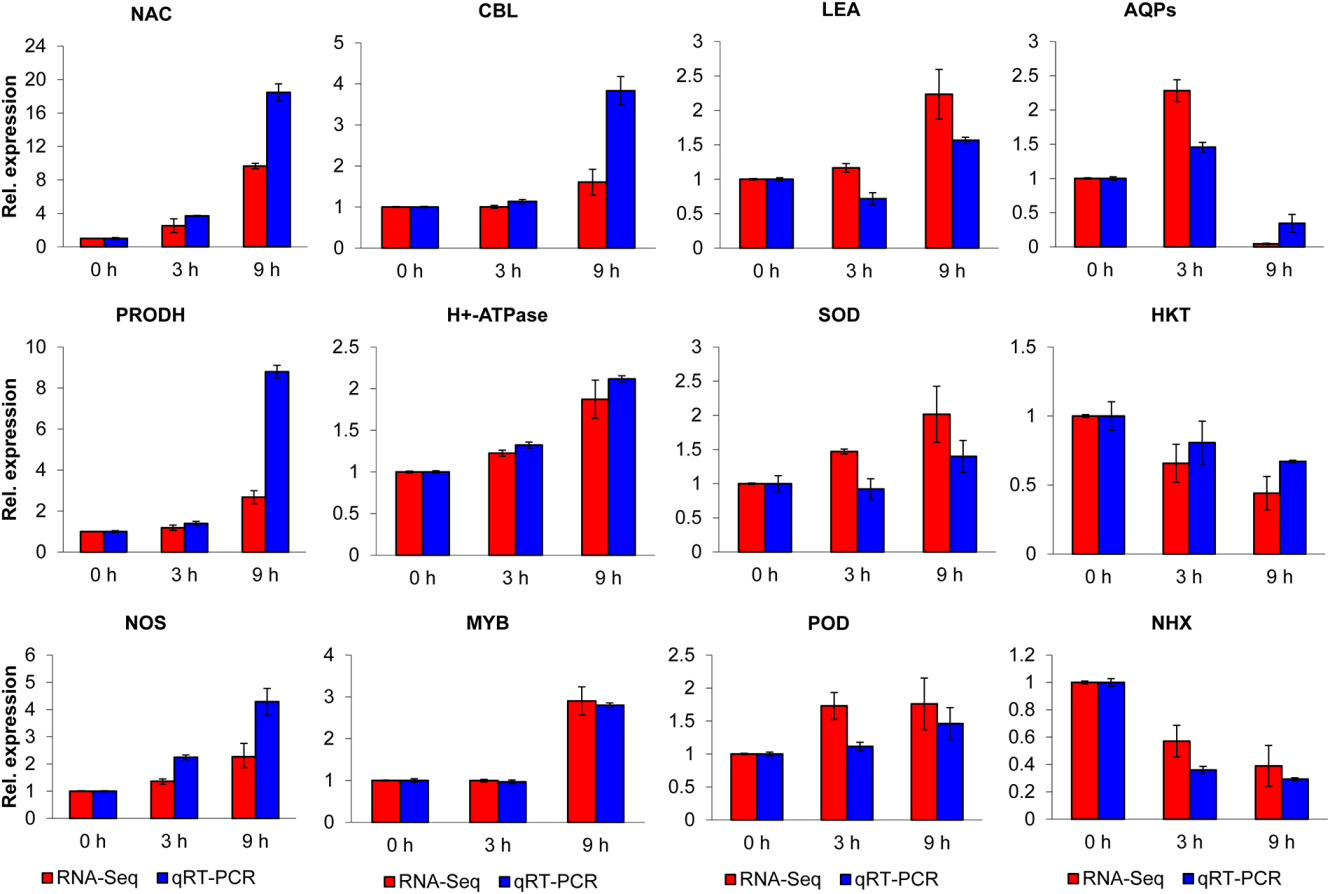
Validation of RNA-seq results by qRT-PCR. Twelve unigenes were randomly selected from among the salt-response-related genes for verification by qRT-PCR. The red bar indicates the RNA-seq results, and the blue bar represents the qRT-PCR outcomes.

To confirm the sequencing data and further understand the expression model of DEGs, 12 unigenes were randomly selected from among the salt-responsive genes for qRT-PCR after 0, 1, 3, 6, 9, 12, 24, and 72 hours of NaCl stress. The primers used for qRT-PCR amplified a single band and had an R^2^ > 0.98 and an amplification efficiency of 90% to 105%. As shown in Fig 6, the gene expression levels detected by Illumina sequencing analysis at 0, 3 and 9 hours were mostly consistent with the qRT-PCR results, excluding the relative expression of three genes (*LEA*, *SOD* and *POD*) at 3 hours. Therefore, our transcriptome sequencing data were generally accurate and reliable for further analyses of salt tolerance in *C*. *chinensis*. The differences were considered to result from the different detection methods used.

**Fig 7:**
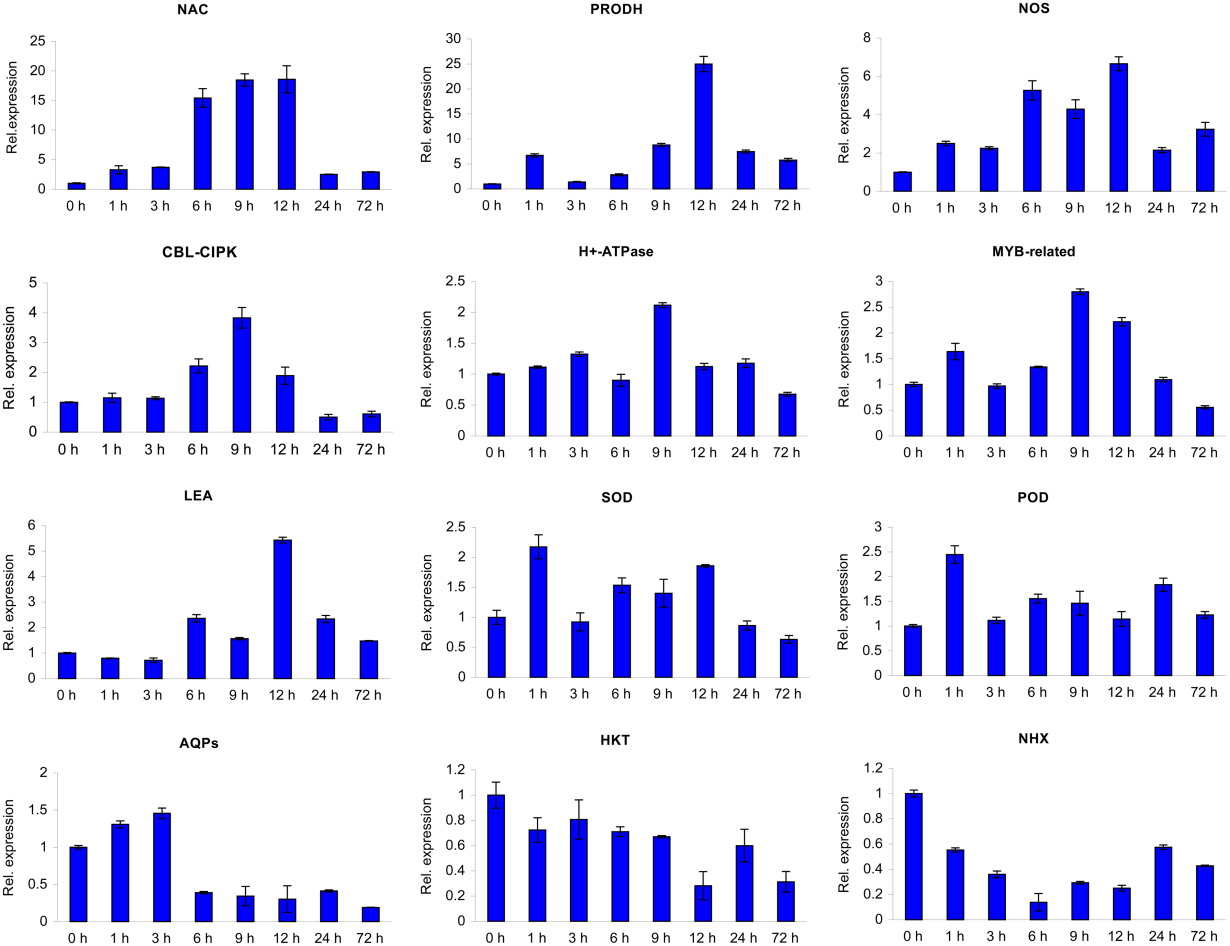
qRT-PCR analysis of 12 DEGs during periods of salt stress. Twelve unigenes used for verification by qRT-PCR were randomly selected from among salt-response-related genes. The qRT-PCR results are the means ± standard deviations (± SDs) of three replicates.

We further studied the expression patterns of these genes during periods of stress. Over the entire 72 hours of salt stress, the 12 validated genes displayed four expression patterns, peaking at 1, 3, 9, and 12 hours (Fig 7). Most genes, including *NAC*, *PRODH*, nitric oxide synthase (*NOS*), *CBL-CIPK*, H^+^-ATPase, *LEAs* and *AQP*s, were up-regulated initially and then down-regulated. By contrast, *HKT* and *NHX* were down-regulated first, slightly up-regulated (below the control), and ultimately down-regulated. Moreover, *MYB*-related, *SOD* and *POD* expression levels were quite complicated, displaying a tendency to fluctuate. The qRT-PCR results illuminated the different regulatory mechanisms of differentially functional genes in salt-responsive processes in *C*. *chinensis*.

## Discussion

Salt stress is one of the most important environmental factors limiting plant growth and development. *C*. *chinensis* is related to *Chrysanthemum*, has ornamental leaves with a dense white tomentum and exhibits high levels of salt tolerance and pest resistance [3, 4]. To assess its salt-response mechanism and explore whether gene resources associated with salt tolerance have practical value for *Chrysanthemum* germplasm innovation, RNA-seq based on NGS can be used to evaluate the transcription levels and expression models of genes from the whole-transcriptome perspective [20].

The proline content, SOD activity, POD activity, and K^+^ /Na^+^ ratio of seedlings can be used as injury indicators in plants exposed to a saline environment [6–8]. According to the results shown in Fig 1, during 72 hours of NaCl stress, most of the indicators had a peak value at 9 hours, which showed significant differences as the control. Regarding short-term stress, the increase was greater at 3 hours than at 1 hour. Therefore, we selected samples exposed to 3 and 9 hours of NaCl stress as the treatments (ST3 and ST9) and 0 hours as the control (ST0) to conduct deep sequencing and related analyses with three biological replicates.

For the *C*. *chinensis* transcriptome, approximately 86.04 G of data were generated and assembled into 163,046 unigenes with an average length of 647 bp. However, only 65,839 unigenes (40.38% of the total) were annotated in the Swiss-Prot database, and the remaining 59.62% had no Swiss-Prot annotations due to the lack of genomic and EST information. The set of unigenes in this study was associated with an extensive range of GO categories, KOG classifications, and KEGG pathways, which indicated that various transcripts were involved in salt-responsive processes in *C*. *chinensis*. GO enrichment analysis further indicated that the differentially expressed transcripts were mainly involved in processes associated with carbohydrate metabolism and biosynthesis (S2 Table and Fig 3), while the KEGG analysis suggested that the genes with differential expression profiles participated in metabolic pathways and environmental information processing, including ‘plant hormone signal transduction’, ‘starch and sucrose metabolism’, ‘biosynthesis of amino acids’, ‘phenylpropanoid biosynthesis’, ‘plant-pathogen interactions’, and ‘carbon metabolism’ (S3 Table and Fig 4).

### Signal transduction pathway analyses

Due to its involvement in photosynthesis, ion homeostasis, antioxidant defense and almost all plant activities, ABA is one of the most important factors in abiotic stress responses [12]. The key components involved in the ABA signaling pathway, such as *PP2C*, *ABF* and *SnRK2s*, were altered in response to salt stress (Table 4 and S4 Table). At 3 hours, *PP2C*-related genes were down-regulated and *SnRK2* was up-regulated to activate the expression of the downstream *ABFs*. At 9 hours, the *PYR/PYL* ABA receptors were differentially expressed, demonstrating 2-fold up- and 1-fold down-regulation. Additionally, 12 differentially expressed *PP2C* genes were down-regulated. *SnRK2*-related genes were consistently up-regulated together with several down-regulated unigenes. In contrast, among 7 *ABRE/ABF*-related genes, 4 were highly expressed, and 3 were up- and then down-regulated during the stress process. *PP2Cs* act negatively and *SnRK2s* act positively in the ABA signal transduction pathway [21, 22]. Therefore, the function of the ABA signaling pathway in *C*. *chinensis* under salt stress is consistent with its roles in *Arabidopsis thaliana* and *Glycine max*.

Calcium acts as an intracellular second messenger that can activate related protein kinases, particularly CaMK, CBLs, and CDPKs, in plants exposed to salinity, resulting in downstream biological reactions [13]. At 3 hours, *CAM*-related Ca^2+^ receptor genes were down-regulated, inhibiting the exchange of inner membrane Ca^2+^ and outer membrane Na^+^ in the ‘phototransduction’ (ko 04744) pathway, while a portion were up-regulated at 9 hours. The *PP3C* gene, which acts downstream in the ‘calcium signaling pathway’ (ko 04020), was down-regulated rather than up-regulated at 3 hours. This gene is also the receptor for calcium signaling in the ‘MAPK signaling pathway’ (ko 04010). Simultaneously, Ca^2+^ passed into the cytoplasm through a non-selective cation channel that is part of the ‘plant-pathogen interaction’ (ko 04626) pathway. On one hand, activated *CAMK* was differentially expressed (6 up-regulated and 2 down-regulated unigenes) and inhibited the formation of ROS by down-regulating the expression of *Rboh*; on the other hand, *CaM*- and *CML*-related genes were largely down-regulated, and the downstream gene *NOA1* was up-regulated, promoting the synthesis of NO, stomatal closure and related stress responses. Additionally, 3 *CaM*-related genes were down-regulated, and 2 were up-regulated in the ‘estrogen signaling pathway’ (ko 04915), which affected the activity of the downstream enzyme NOS3. Additionally, *CDPKs* are the largest group in the Ca^2+^ receptor family in plant cell-mediated Ca^2+^ signal transduction processes, including stress responses [23]. Ca^2+^ is also a key messenger in many signal transduction pathways in *C*. *chinensis* under salt stress, consistent with the findings of Knight et al. [24]. The details of the related DEGs are shown in S5 Table.

*CBLs* regulate the Na^+^ balance in the cytoplasm via the CBL-CIPK combination by activating the Na^+^/H^+^ antiporter SOS1 and *NHX* expressed in the SOS signal transduction pathway [25]. The SOS pathway is a major means by which ionic homeostasis is regulated by preventing Na^+^ entry into the cell, relieving potassium starvation, causing the outflow of Na^+^, and segregating Na^+^ into the vacuole [14]. The main genes involved in the process include *SOS1*, *SOS2*, *SOS3*, *SOS4*, *AKT1*, *NHX1*, *KUP*, *HKT1* and *CBL/CIPK* [26, 27]. Only 3 of 24 *CBL/CIPK*-related DEGs in the *C*. *chinensis* transcriptome were up-regulated, one of which was further analyzed by qRT-PCR. The variation in relative expression with persistent stress for 72 hours revealed that this unigene (c44415_g1) continued to be regulated under NaCl stress for up to 9 hours (Fig 7), supporting its induction in response to salt stress in *C*. *chinensis* [25]. H^+^-ATPase-related genes were either up-regulated (11) or down-regulated (9) at 9 hours, and, like the *CBL* unigene, one of the two plasma H^+^-ATPases (c40465_g1) was validated for its up-regulation to 9 hours (Fig 7). *AKT*- and *HKT*-related DEGs were down-regulated at 9 hours. The down-regulation of *NHX*-related genes (c49840_g1) in *C*. *chinensis*, indicating Na^+^ isolation, did not occur in response to salt stress at the transcript level, while the down-regulation of the Na^+^ transporter *HKT* (c40570_g2) could prevent Na^+^ entry into the cytoplasm [28]. However, most related *SOS* DEGs were not responsive or were down-regulated at the level of transcription, which is consistent with Sharma et al. [29] and has been proposed to explain the regulation of *SOS* genes primarily at the posttranslational level [30]. The details of the related DEGs are shown in S6 Table.

### Responses of compatible solutes

Genes related to the metabolism of arginine, proline, glycine, starch and sucrose, as well as genes related to flavonoid biosynthesis, were differentially expressed at distinct time points (0, 3, and 9 hours) (Table 3). As an important osmotic-adjusting substance, proline accumulates to enhance plant tolerance to adverse conditions [6]. Interestingly, in *C*. *chinensis*, the accumulation of proline was not significant, and its biosynthesis was mainly from ornithine instead of glutamic acid, which also indicated a high level of nitrogen [31]. However, the up-regulation of *PRODH2* (c30247_g1) promotes proline accumulation [32], and the trend toward up-regulation shown in Fig 7 indicates that it may function in the salinity response processes in *C*. *chinensis*. On one hand, ornithine is transformed into putrescine via the expression of ornithine decarboxylase (*ODC*)-related genes. On the other hand, the activation of NOS aided the synthesis of arginine, which was inhibited to generate putrescine via the down-regulation of arginine decarboxylase (*ADC*)-related genes, which further promoted the production of NO. Concomitantly, the up-regulation of polyamine oxidase (*PAO*)-related genes helped produce putrescine from spermine and spermidine. Feedback regulation exists between polyamine and NO during plant responses to salt stress [33]. On one hand, NO is induced as a signal by polyamine to enhance the activity of H^+^-ATPase. On the other hand, high NO content changed the ratio of the three free polyamines (putrescine, spermidine, and spermine), removed free radicals, and balanced the K^+^/Na^+^ ratio to adjust the salinity. Studies have shown that the polyamine contents increased during short periods of stress and decreased or rarely changed over long periods of stress, indicating that polyamines changed with different durations of stress [34–36]. Additionally, the qRT-PCR results showed that the relative expression of the *NOS* unigene (c31403_g1) remained higher than that in the control (Fig 7). Therefore, we propose that proline and polyamine potentially functioned initially, while the NO signaling pathway was primarily responsive to salt stress in *C*. *chinensis*. The details of the differential expression of related genes are shown in S7 Table.

### Genes encoding functional proteins

The LEA proteins in many species have high expression levels under stress conditions such as drought, UV radiation, high salt, low temperatures, ethylene, and ABA [37]. Transcripts encoding a total of 13 LEA proteins were detected in the *C*. *chinensis* transcriptome, 5 of which were relatively enriched for ‘response to stress’ GO terms (S8 Table). One up-regulated unigene (c27773_g1) was homologous to *TcLEA*, which has an unknown biological function [38], but qRT-PCR indicated persistent up-regulation for 12 hours (Fig 7). *CsLEA5* is induced by salt, drought and heat stress [39], but the 2 homologous genes in *C*. *chinensis* were down-regulated. The overexpression of *SiLEA* enhances the salt tolerance of transgenic *Arabidopsis thaliana* [40]. Additionally, the homolog displayed high constitutive expression. *LEAs* can efficiently enhance the drought and salt tolerance of transgenic plants [41], although their mechanism in *C*. *chinensis* requires further analysis.

*AQPs* are a family of integral membrane proteins that facilitate the transport of small molecules such as water, small uncharged solutes, and gases across biological membranes [42]. A total of 73 *AQP*-related genes were detected in the *C*. *chinensis* transcriptome (Table 4 and S8 Table). According to the KO annotation, 28 and 12 of these genes were *PIPs* and *TIPs*, respectively, each with 2 constitutive expression levels; 12 were *NIPs* that included 5 down-regulated and 2 constitutively expressed DEGs; 5 were *SIPs*, 4 of which were constitutively expressed; and 15 were others. *TIPs* and PIPs included more DEGs, with 2 and 3, respectively, up- and then down-regulated and 3 and 10, respectively directly down-regulated at 3 hours. In general, *AQP* expression is first down-regulated to reduce membrane water conductivity to prevent water deficiency caused by osmotic stress under salinity, and then it recovers to or up to the level observed prior to stress with the accumulation of osmotic substances [43]. The overexpression of *AQPs* can also enhance the salt tolerance of *Oryza sativa* and *Arabidopsis thaliana* [44, 45]. In our study, one *PIP* unigene (c48016_g1) was validated through qRT-PCR to be up-regulated during the first 3 hours and then sharply down-regulated during the 72-hour period of NaCl stress (Fig 7). We hypothesized that the down-regulation of unigene c48016_g1 from 6 to 72 hours might be a reaction to osmotic stress [43]. Based on the variation in related DEGs, we presume that *TIPs* and *PIPs* play important roles in the salt response of *C*. *chinensis*. However, their functional mechanisms should be further clarified through additional research.

Salt stress induces a series of oxidation-reduction reactions that lead to the accumulation of excessive ROS in plants. ROS such as O_2_^-^ are first transformed into H_2_O_2_ by SOD and then eliminated by other antioxidant enzymes [46]. The accumulation of ROS would result in the expression of Cu/Zn-SOD genes, Fe-SOD genes and Mn-SOD genes. Additionally, a reduction in one type of SOD gene will promote the expression of the other types of SOD genes [47]. Based on the KO annotation, one Cu/Zn-SOD unigene (c43559_g1) was down-regulated, and one Fe/Mn-SOD unigene (c31882_g1) was up-regulated; both were differentially expressed and are part of the peroxisome pathway (ko04146). SODs play a role when plants are under adverse conditions, and different types of SOD genes are adjusted [47]. The glutathione-ascorbate cycle involves several antioxidant metabolites and enzymes. Glutathione (GSH) acts as a redox sensor and is regenerated from its oxidized form, oxidized glutathione (GSSG), by the action of glutathione reductase (GR), glutathione peroxidase (GPX) or glutathione S-transferase (GST) to maintain lower levels of ROS [48, 49]. Ascorbate peroxidase (APX) scavenges H_2_O_2_ by transferring ascorbate (ASC) to monodehydroascorbate (MDHA). In the *C*. *chinensis* transcriptome, many antioxidant enzymes were differentially expressed (Table 4 and S8 Table): 2 *GPX*-related genes were down-regulated to prevent the oxidation of GSH; 11 differentially expressed and 11 highly expressed GST-related genes were detected; *APX* followed the up- and then down-regulated model; and POD-related genes were differentially expressed in the ‘phenylpropanoid biosynthesis’ (ko 00940) pathway, with 8 up-regulated and 12 down-regulated genes and most unigenes being relatively highly expressed, similar to *GST* genes. Although the relative expression of the SOD (c31882_g1) and POD (c49195_g3) unigenes fluctuated during the 72 hours of NaCl stress (Fig 7), the differential expression of certain antioxidant enzymes indicated that the ROS scavenging system was responsive to salt stress in *C*. *chinensis*. However, uncovering the accurate functional mechanism requires further research.

### Responses of transcription factors

In the *C*. *chinensis* transcriptome, among transcription factors, the differentially expressed *ERF*, *bZIP*, *bHLH*, and *MYB*-related genes tended to be down-regulated. In contrast, most WRKY and DREB genes showed the opposite trend, with 10 *WRKY* and 5 *DREB* genes up-regulated and 2 *WRKY* and 3 *DREB* genes down-regulated. Relatively highly expressed genes accounted for 29% of all detected *WRKY* genes, 20% of *bHLH* genes, 14% of *NAC* genes, and 12% each of *ERF* and *MYB* genes. The details are shown in S9 Table. Three up-regulated genes were homologous to *CmWRKY10* (*AtWRKY65*), which is expressed under normal conditions in the *Chrysanthemum* cultivar ‘Jinba’ and has an unknown biological function [50]. The other 6 up-regulated *WRKY* genes were homologous to *AtWRKY48*, *AtWRKY57*, *AtWRKY4*, *AtWRKY20*, *AtWRKY26*, and *AtWRKY27*, and most were induced by abiotic stress [51–54]. In contrast, genes homologous to *CmWRKY12*/*AtWARK17* and *CmWRKY11*/*AtWARK70* were down-regulated, despite the former being up-regulated and the latter being up- and then down-regulated under stress. Additionally, in addition to *CmWRKY4*, all 9 highly expressed *Chrysanthemum* homologous genes were up-regulated [50]. *WRKY*-family genes were considered important transcription factors in response to salt in *C*. *chinensis*. *NAC* genes were detected and included 16 DEGs and 14 relatively highly expressed unigenes (S9 Table). The two homologs of *DgNAC* (1 down-regulated and 1 highly expressed) were shown to enhance the salt tolerance of *Nicotiana tabacum* [55]. One up-regulated *NAC* gene (c33036_g1) was homologous to *AtNAC055*, which was induced by salt stress and, based on overexpression, confirmed to promote the up-regulation of other tolerance genes [56]. This *NAC* unigene (c33036_g1) was also shown by qRT-PCR to be up-regulated during 12 hours of NaCl stress, indicating that it may respond to salt stress in *C*. *chinensis*. Based on the data presented in S9 Table, the up-regulated *bHLH*-family genes in *C*. *chinensis* were most homologous to *AtbHLH25*, *AtbHLH130*, *AtbHLH63*, and *AtbHLH66*, which are related to cell metabolism and growth regulation [57, 58]. In contrast, the salt-induced *AtbHLH112* and *AtbHLH28* homologs were down-regulated [59]. This finding indicated that *bHLH*-family genes may function in growth regulation during salt stress. There were 6 *MYB*-family genes in *C*. *chinensis* (3 up-regulated, 2 down-regulated and 1 highly expressed), which were homologous to those in *Chrysanthemum* (S9 Table). One of these genes was validated by qRT-PCR. The results showed that this *MYB* unigene (c47164_g1) was up-regulated rapidly during the first hour of stress, then gradually increased in expression until it peaked at 9 hours; the tendency toward up-regulation indicated that this unigene functioned positively during NaCl stress (Fig 7). *CmMYB2* was induced by drought, salt, cold and high levels of ABA, possibly functioning in salt and drought tolerance as well as in the control of flowering [60]. The overexpression of *AtMYB44* could inhibit the expression of *PP2C* genes and then negatively regulate the ABA signaling pathway to enhance the tolerance of transgenic *Arabidopsis thaliana* [61]. There were 7 *DREB* family genes in *C*. *chinensis* (3 that were down- and then up-regulated) that were homologous to those in *Chrysanthemum* (S9E Table). Heavy salt stress increased the expression of *DmDREBa*, which has been reported to enhance the tolerance of *Nicotiana tabacum* during cold, drought and salt stress [62]. Two other up-regulated *DREB* genes in *C*. *chinensis* were homologous to *AtDREB3* and *AtDREB2A*, which regulate salt and dehydration stress at the transcript level [63]. Therefore, these transcription factors are valuable for further analyses of their molecular functions in *C*. *chinensis* under salt stress.

## Conclusions

This study provides comprehensive information about the transcriptome of the non-model plant *C*. *chinensis* under salt stress. In conclusion, our results show that multiple genes and pathways, such as *AQP*-, *WRKY*-, *MYB*-, and *AP2/EREBP*-family genes and NO, plant hormone, and calcium signaling pathways, are involved in salt responses. These findings represent a first step toward illuminating the molecular mechanisms underlying salt tolerance in *C*. *chinensis*, and they also provide abundant genomic resources and new candidate genes for studies on tolerant germplasm innovation and molecular biology in the related genus *Chrysanthemum*.

## Materials and methods

### Plant materials

Previous experiments conducted by our research team indicate that *C*. *chinensis* is valuable for salinity studies of specific leaf structures and salt tolerance during exposure to high concentrations of NaCl (360 mM). Therefore, further analyses were conducted at the molecular level to explore probable influential factors. The plant materials used in this research were collected from Fujian Province and preserved in the *Chrysanthemum* Germplasm Resource Preservation Center, Beijing Forestry University, China. The seedlings were cultivated in a mixture of perlite and vermiculite (1:1) in the greenhouse with day and night temperatures of 25 ± 5°C and 18 ± 2°C, respectively. Shoot cuttings of *C*. *chinensis* were rooted and grown in a sand bed. Rooted seedlings at the 6–8 leaf stage were selected and transplanted into 170 mL plastic pots filled with clean quartz sand and irrigated with Hoagland nutrient solution after one month. After one week of recovery, the plants were treated with 360 mM NaCl for a duration of 0 (CK), 1, 3, 6, 9, 12, 24 and 72 hours according to a previous experiment performed by our research team. The plants were randomly divided into equal groups such that each treatment included three biological replicates.The leaves were harvested and snap frozen in liquid nitrogen. The frozen samples were preserved at -80°C for subsequent experiments.

### Measurement of physiological parameters

Physiological parameters, including proline content, POD activity, SOD activity and K^+^/Na^+^ ratio, were measured to determine the key point of the *C*. *chinensis* response to salt stress. All measurements were performed using Li's method [64] with modifications and according to the manufacturers’ instructions. Three treatments, including the control, each with three biological replicates, were selected for high-throughput sequencing.

To measure the proline content, fresh leaves (0.2 g) were cut into pieces and put into tubes, and 5 mL of an aqueous 3% sulfosalicylic acid solution was added. The mixture was exposed to boiling water for 10 minutes, 2 mL of the mixture was put into clear tubes, 2 mL of acetic acid and 2 mL of acidic ninhydrin were added and mixed, and the tubes were placed in boiling water for 30 minutes. After cooling the mixture, 4 mL of toluene was added into the reaction solution, and the solution was allowed to stand until the extraction completed. The upper liquor was centrifuged at 3000 r/min for 5 minutes and tested at 520 nm to obtain the absorbance values. Proline contents were calculated using a standard curve.

For ion measurements, mature leaf samples were washed and heated at 105°C for 25 minutes. After the samples were dried to a constant weight at 70°C, the dry weight of the samples was measured. The samples were then ground and put into a dryer for storage. Fifty milligrams of dry sample was added to a tube, 20 mL of water was added, and the sample was vortexed. The samples were filtered into 25 mL volumetric flasks after incubation in a boiling water bath for 1.5 h. The K^+^ and Na^+^ contents of the nutrient solutions from each treatment were measured using flame atomic absorption spectrometry (Varian 220, Perkin Elmer Co.).

To measure the activity of SOD and POD, a 0.5 g leaf sample was harvested at the end of each treatment and extracted according to the method of Li [64]. The leaf material was homogenized in 10 mL of 0.05 mol L^-1^ phosphate buffer solution (pH 7.8) at 4°C. The homogenate was filtered and centrifuged at 10,000 r/min for 20 min at 4°C. The supernatant was maintained at 4°C to measure enzyme activity. SOD activity was assayed using the photochemical NBT method, which utilized 13 mM L-methionine, 75 μM NBT, 10 μM EDTA-Na2 and 2 μM riboflavin. The reduction of NBT was monitored at 560 nm, and an inhibition curve was constructed using various volumes of extract. One unit of SOD activity was defined as that sufficient to inhibit the photo-reduction of NBT by 50%. POD activity was determined in a reaction mixture containing 2.9 mL of 0.05 mol L^-1^ phosphate buffer solution (pH 7.8), 1 mL of 2% H_2_0_2,_ 1 mL of 0.05 mol L^-1^ guaiacol, and 0.1 mL supernatant. The two substrates were freshly prepared just before use. The system was assayed by measuring the change in absorbance at 470 nm, and time was counted immediately. The activity was calculated by measuring the ratio at 470 nm, and a change of 0.01 per minute was defined as one unit of activity.

### RNA extraction, library preparation and sequencing

Total RNA was extracted using an EASYspin Plus Plant RNA Kit (Aidlab Biotech, Beijing, China) according to the manufacturer’s instructions. Extracted RNA was assessed for quality and quantity using a NanoPhotometer spectrophotometer^®^ (IMPLEN, CA, USA), a Qubit^®^ RNA Assay Kit with a Qubit^®^ 2.0 Fluorometer (Life Technologies, CA, USA) and an RNA Nano 6000 Assay Kit with an Agilent Bioanalyzer 2100 system (Agilent Technologies, CA, USA). A total of 3 μg RNA per sample was used as input material for the RNA sample preparation. Sequencing libraries were generated using an NEBNext^®^ Ultra^™^ RNA Library Prep Kit for Illumina^®^ (NEB, USA) following the manufacturer’s recommendations, and index codes were added to attribute sequences to each sample. Briefly, qualified samples were used for mRNA purification using poly-T-oligo-conjugated magnetic beads and were then fragmented into small pieces. After the first-strand cDNA was synthesized using random hexamers and M-MuLV Reverse Transcriptase (RNase H-), second-strand cDNA synthesis was performed using DNA polymerase I and RNase H. The remaining overhangs were converted into blunt ends via exonuclease/polymerase activities. After purification using an AMPure XP system (Beckman Coulter, Beverly, USA), using EB buffer, the double-stranded cDNAs were resolved for size selection and adaptor ligation. PCR was then performed with Phusion High-fidelity DNA polymerase, universal PCR primers and Index (X) Primer. Finally, the PCR products were purified (AMPure XP system), and the library quality was assessed using the Agilent Bioanalyzer 2100 system. The clustering of the index-coded samples was performed on a cBot Cluster Generation System using a TruSeq PE Cluster Kit v3-cBot-HS (Illumina) according to the manufacturer’s instructions. After cluster generation, the libraries were sequenced using the Illumina HiSeq platform, and paired-end reads were generated.

### Assembly, gene expression and annotation

Low-quality reads were removed using an in-house Perl script. Clean data (clean reads) were obtained by removing reads containing adapters, reads containing poly-N sequences, and reads of low quality from the raw data. Simultaneously, the Q20, Q30, GC content and sequence duplication level of the clean data were calculated. All downstream analyses were based on clean, high-quality data (PhredScore > 20). Transcriptome assembly was accomplished using Trinity software (http://trinityrnaseq.sourceforge.net/) with a min_kmer_cov set to 2 by default and all other parameters set to their defaults [65]. Clean data were mapped back onto the assembled transcriptome. The read count for each gene was obtained from the mapping results. The gene expression levels were estimated by RSEM [66] for each sample and calculated by RNA-seq quantification analysis in terms of FPKM [67]. Gene function was annotated using the following databases: Nr (NCBI non-redundant protein sequences); Nt (NCBI non-redundant nucleotide sequences); Pfam (protein family); KOG/COG (Clusters of Orthologous Groups of proteins); Swiss-Prot (a manually annotated and reviewed protein sequence database); KO (KEGG orthologue database) and GO (Gene Ontology).

### Differential expression analysis

For the samples with biological replicates, the differential expression analysis of two conditions/groups was performed using the DESeq R package (1.10.1) [68]. DESeq provides statistical routines for determining differential expression in digital gene expression data using a model based on a negative binomial distribution. The resulting P-values were adjusted using Benjamini and Hochberg’s approach for controlling the false discovery rate. Genes with an adjusted P-value (padj) < 0.05 as determined by DESeq were considered differentially expressed. GO enrichment analysis of the DEGs was implemented in the GOseq R package-based Wallenius non-central hyper-geometric distribution [69], which can adjust for gene length bias in DEGs. The relative transcript abundance was obtained by including the *C*. *chinensis* EF1-α gene as the reference and was based on the 2^-ΔΔCt^ method [70]. KEGG [71] is a database resource for understanding the high-level functions and utilities of the biological system, such as the cell, the organism and the ecosystem, from molecular-level information, especially large-scale molecular datasets generated by genome sequencing and other high-throughput experimental technologies (http://www.genome.jp/kegg/). We used KOBAS [72] software to test the statistical enrichment of DEGs in the KEGG pathways.

### Real-time quantitative PCR (qRT-PCR)

Total RNA was extracted from leaf samples obtained as described above. After treatment with DNase, the total RNA was subjected to reverse transcription to produce cDNA using a reverse transcription system. Real-time RT-PCR was conducted using SYBR Green (TaKaRa, Japan) and a PikoReal real-time PCR system. Each reaction was performed in a total volume of 10 μL, which included of 2 μL of first-strand cDNA as a template. The amplification program was as follows: 1 minute at 95°C and 40 cycles of 15 seconds at 95°C, 15 seconds at 60°C and 45 seconds at 72°C. Gene-specific primers (S1 Table) were designed for the relative quantification of each gene. Three biological replicates were performed for each sample. The relative transcript abundance was determined by including the *C*. *chinensis* EF1-α gene as a reference and was based on the 2^-ΔΔCt^ method [70].

## Statistical analysis

All measurements were subjected to an one-way ANOVA to compare the mean values using the SPSS 19.0 statistical package at the p< 0.05 level. Duncan post hoc tests were performed to compare the mean values in case of significant differences.

## Supporting information

S1 Table Primers used in qRT-PCR in *Crossostephium chinensis*. (XLSX)

S2 Table Details of GO enrichment with padj < 0.05. (XLSX)

S3 Table Details of KEGG enrichment with padj < 0.05. (XLSX)

S4 Table DEGs involved in the ABA signaling pathway. (XLSX)

S5 Table DEGs involved in the Ca^2+^ signaling pathway. (XLSX)

S6 Table DEGs involved in the SOS signaling pathway. (XLSX)

S7 Table DEGs involved in compatible solutes. (XLSX)

S8 Table DEGs involved in functional proteins. (XLSX)

S9 Table Differentially expressed transcription factor genes. (XLSX)

S1 Fig Length distribution of transcripts and unigenes. (TIF)

S2 Fig Gene Ontology (GO) functional annotation of transcripts. The results are summarized in three main categories: biological process, cellular component, and molecular function. The y-axis indicates the number of genes in a category. A total of 53,368 unigenes were assigned to GO terms.
(TIF)

S3 Fig EuKaryotic Orthologous Groups (KOG) classifications in *C*. *chinensis*. A total of 34,183 sequences with KOG classifications within the 26 categories are shown. The y-axis indicates the percentage of genes in a category.
(TIF)

S4 Fig Categorization of *C*. *chinensis* unigenes in KEGG biochemical pathways. Genes are classified into five categories according to the KEGG pathway in which they participate: A, cellular processes; B, environmental information processing; C, genetic information processing; D, metabolism; E, organismal systems. A total of 37,417 unigenes were assigned to KEGG terms. The x-axis indicates the percentage of genes in a category.
(TIF)
