# Supplementary figures and images for: Transcriptome analysis of Crossostephium chinensis provides insight into the molecular basis of salinity stress responses
Source: PLoS One. 2017 Nov 13;12(11):e0187124. doi: 10.1371/journal.pone.0187124 (PMC5683599; doi:10.1371/journal.pone.0187124)

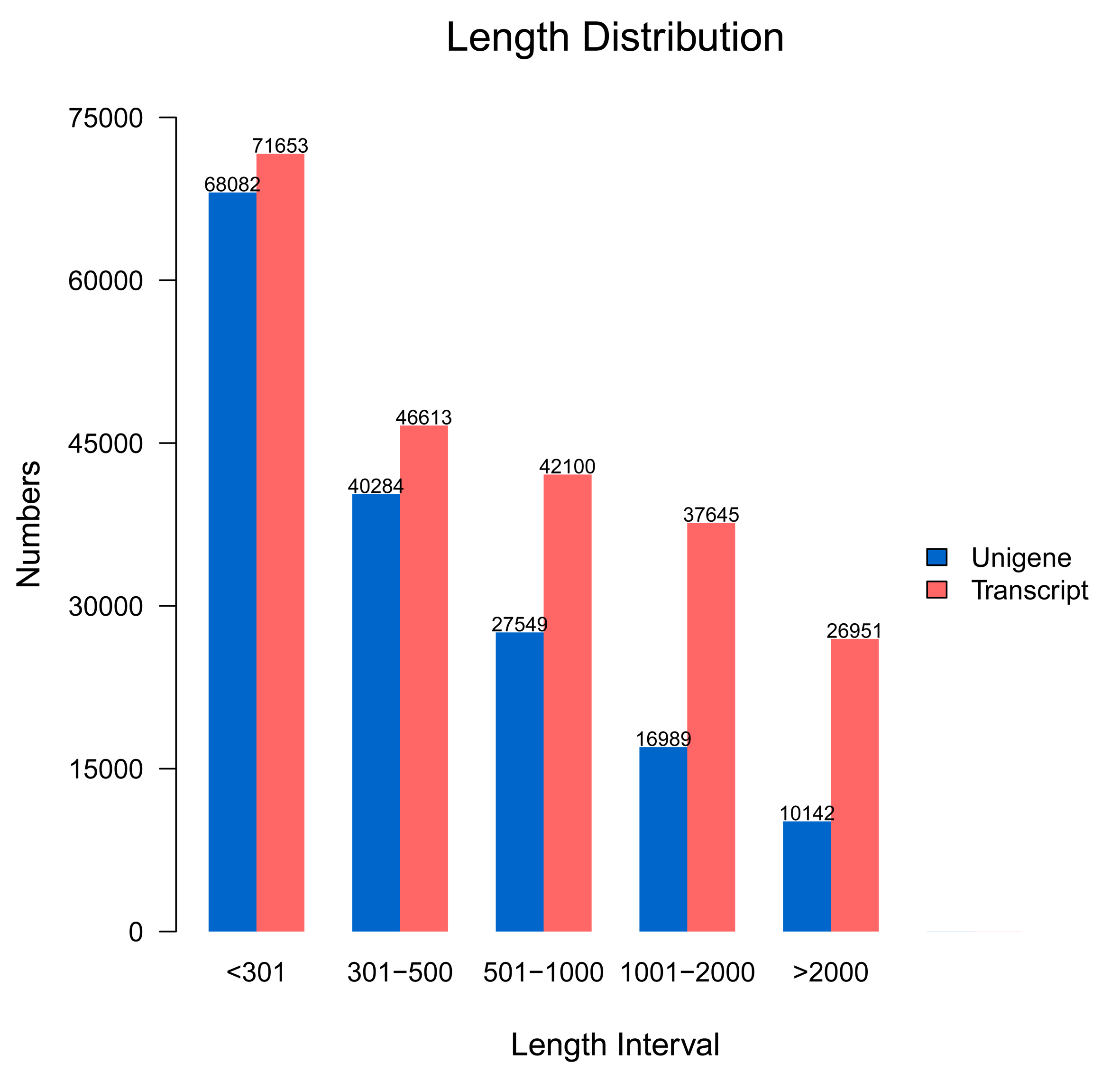

Supplement: S1 Fig — (TIF) [file pone.0187124.s010.tif]

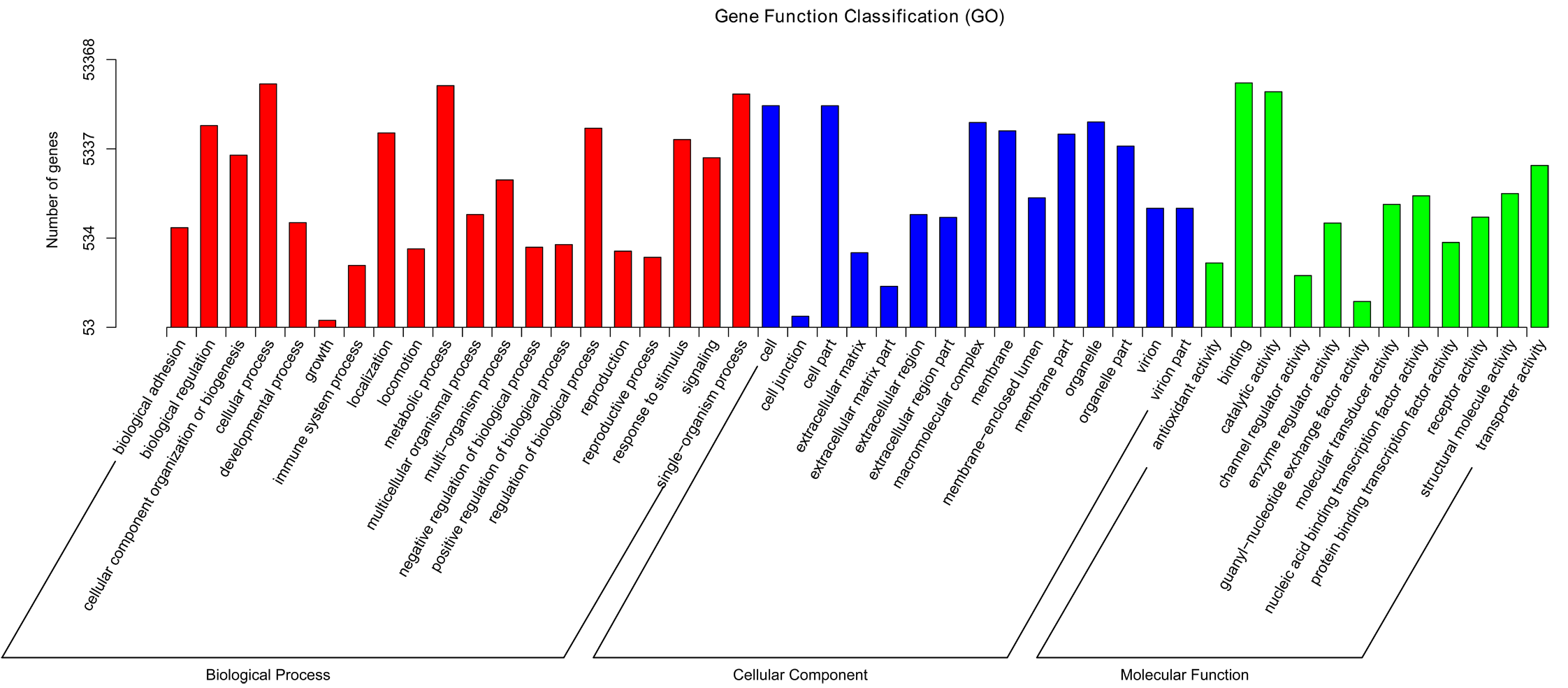

Supplement: S2 Fig — The results are summarized in three main categories: biological process, cellular component, and molecular function. The y-axis indicates the number of genes in a category. A total of 53,368 unigenes were assigned to GO terms. (TIF) [file pone.0187124.s011.tif]

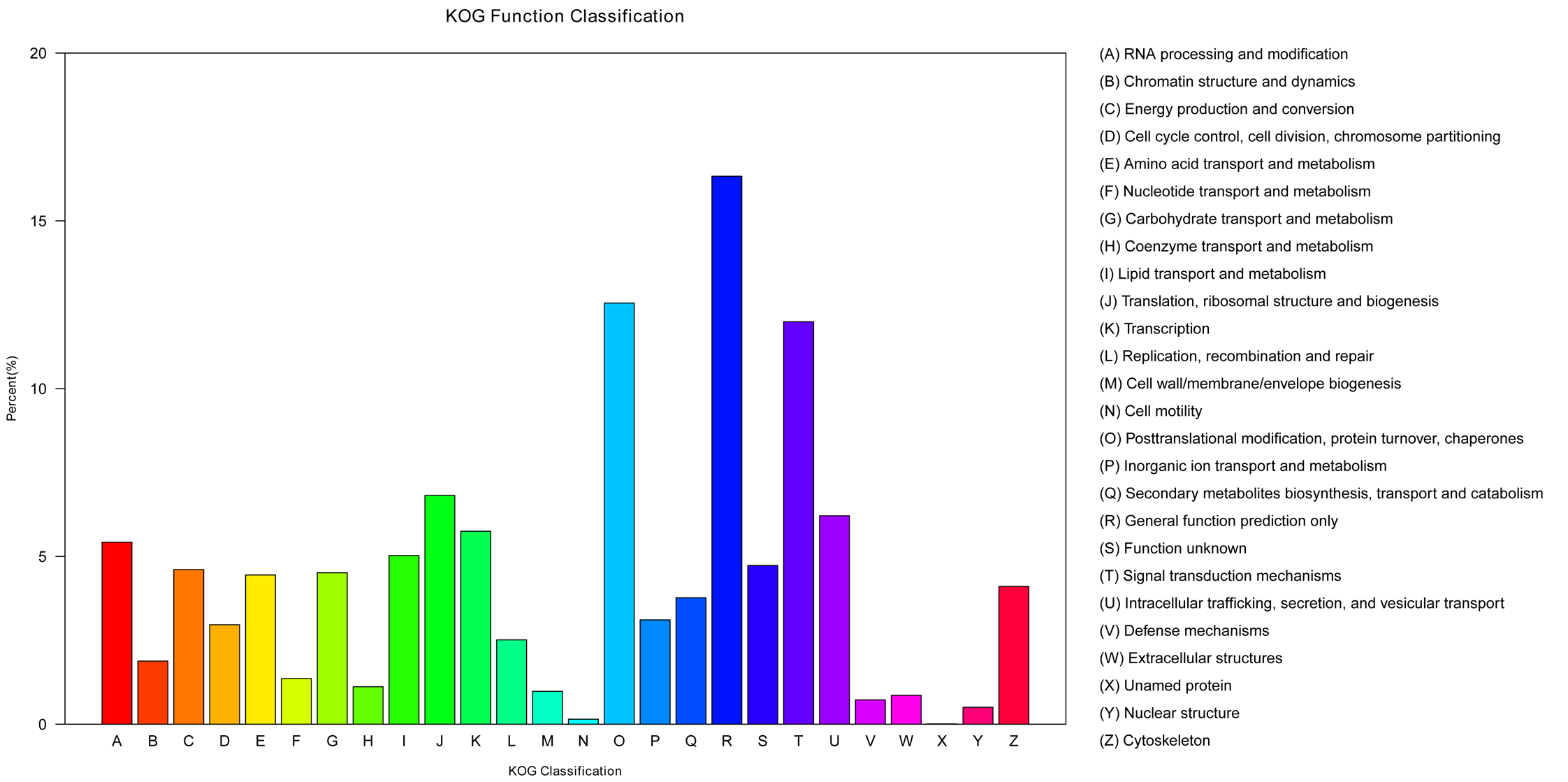

Supplement: S3 Fig — A total of 34,183 sequences with KOG classifications within the 26 categories are shown. The y-axis indicates the percentage of genes in a category. (TIF) [file pone.0187124.s012.tif]

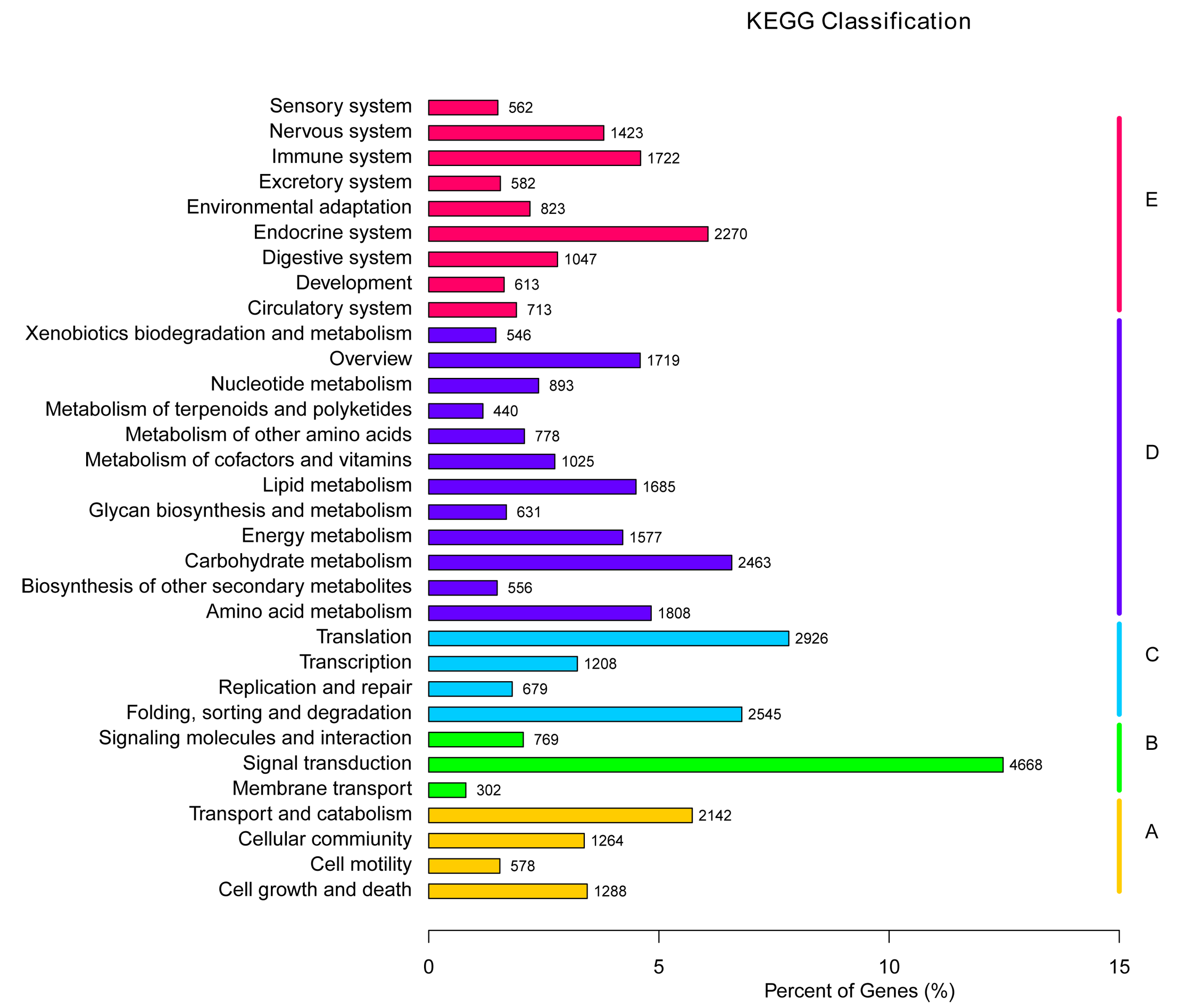

Supplement: S4 Fig — Genes are classified into five categories according to the KEGG pathway in which they participate: A, cellular processes; B, environmental information processing; C, genetic information processing; D, metabolism; E, organismal systems. A total of 37,417 unigenes were assigned to KEGG terms. The x-axis indicates the percentage of genes in a category. (TIF) [file pone.0187124.s013.tif]
